# Phosphoinositide 3′-Kinase γ Facilitates Polyomavirus Infection

**DOI:** 10.3390/v12101190

**Published:** 2020-10-20

**Authors:** Paul Clark, Gretchen V. Gee, Brandon S. Albright, Benedetta Assetta, Ying Han, Walter J. Atwood, Daniel DiMaio

**Affiliations:** 1 Department of Genetics, Yale School of Medicine, P.O. Box 208005, New Haven, CT 06520-8005, USA; paul.clark@yale.edu (P.C.); bsa9878@gmail.com (B.S.A.); yhan811@gmail.com (Y.H.); 2 Molecular Biology, Cell Biology and Biochemistry, Brown University, Box G-E431, Lab of Molecular Medicine, 70 Ship Street, Providence, RI 02912, USA; gretchen.gee@UMASSMED.edu (G.V.G.); benedetta_assetta@brown.edu (B.A.); 3 Department of Therapeutic Radiology, Yale School of Medicine, P.O. Box 208040, New Haven, CT 06520-8040, USA; 4 Department of Molecular Biophysics & Biochemistry, Yale University, P.O. Box 208024, New Haven, CT 06520-8024, USA; 5 Yale Cancer Center, P.O. Box 208028, New Haven, CT 06520-8028, USA

**Keywords:** JC virus, polyomavirus, SV40, progressive multifocal leukoencephalopathy, PI3Kγ, PML

## Abstract

Polyomaviruses are small, non-enveloped DNA tumor viruses that cause serious disease in immunosuppressed people, including progressive multifocal leukoencephalopathy (PML) in patients infected with JC polyomavirus, but the molecular events mediating polyomavirus entry are poorly understood. Through genetic knockdown approaches, we identified phosphoinositide 3′-kinase γ (PI3Kγ) and its regulatory subunit PIK3R5 as cellular proteins that facilitate infection of human SVG-A glial cells by JCPyV. PI3Kα appears less important for polyomavirus infection than PI3Kγ. CRISPR/Cas9-mediated knockout of PIK3R5 or PI3Kγ inhibited infection by authentic JCPyV and by JC pseudovirus. PI3Kγ knockout also inhibited infection by BK and Merkel Cell pseudoviruses, other pathogenic human polyomaviruses, and SV40, an important model polyomavirus. Reintroduction of the wild-type *PI3Kγ* gene into the PI3Kγ knock-out SVG-A cells rescued the JCPyV infection defect. Disruption of the PI3Kγ pathway did not block binding of JCPyV to cells or virus internalization, implying that PI3Kγ facilitates some intracellular step(s) of infection. These results imply that agents that inhibit PI3Kγ signaling may have a role in managing polyomavirus infections.

## 1. Introduction

Human polyomaviruses establish lifelong, persistent, usually asymptomatic infections in most humans [1,2]. Several polyomaviruses, including JC polyomavirus (JCPyV), BK polyomavirus (BKPyV), and Merkel cell polyomavirus (MCPyV), cause serious disease in immunocompromised patients. JCPyV normally replicates in the kidney and is the causative agent of progressive multifocal leukoencephalopathy (PML), a rapidly progressive, often fatal central nervous system demyelinating disease for which there are no effective treatments [2,3,4,5,6]. PML is a significant cause of morbidity and mortality in AIDS patients, and is considered an AIDS-defining illness [3]. PML can also be a fatal complication in a small fraction of patients receiving immunomodulatory antibodies as treatment for multiple sclerosis and other disorders [7,8,9,10,11,12]. In contrast to JCPyV, BKPyV causes pathology primarily localized to the reno-urinary tract, including polyomavirus-associated nephropathy (PyVAN) in kidney transplant recipients and hemorrhagic cystitis in bone marrow transplant recipients [13,14]. PyVAN is associated with graft dysfunction and premature graft loss in more than 50% of cases in which BKPyV is actively replicating in the organ [15,16,17]. Although JCPyV also replicates in the kidney, few cases of nephropathy are attributed to JCPyV [18,19,20,21], and when it occurs, JC PyVAN is less severe than BK PyVAN and is associated with a better prognosis [22,23]. The reasons for these differences are unknown.

Long-term, persistent MCPyV infection in immunodeficient individuals is associated with the development of Merkel Cell Carcinoma, an aggressive and often fatal form of skin cancer that appears to arise from neuroendocrine cells that reside in the skin [24,25,26]. The mechanism of MCPyV carcinogenesis involves viral DNA integration into the cell genome and continued expression of the polyomavirus transforming proteins [27,28,29].

JCPyV, BKPyV, and MCPyV have distinct tissue and host cell tropisms, presumably due to several factors, including the availability of cell-surface receptors on the host cell; the presence or absence of specific cell proteins that mediate intracellular trafficking, transcription, and replication of the virus; and the ability of infected cells and hosts to mount effective immune responses to the infecting virus. Both JCPyV and BKPyV trigger innate immune signaling mechanisms dependent on the cell type that is infected. For example, BKPyV elicits an innate immune response in bladder endothelial cells, resulting in control of the virus in the bladder but not in renal proximal tubule epithelial cells [30,31]. Understanding the specific cell types that are targets of acute and persistent infection by polyomaviruses is critical to our understanding of how these viruses switch from a benign persistent state to an acute highly pathogenic state.

The polyomaviruses are non-enveloped DNA viruses with capsids comprised of 72 pentamers of the major capsid protein (VP1) as well as the minor capsid proteins (VP2/3) buried under VP1 [32]. For successful infection, these viruses must deliver their double-stranded DNA genomes to the cell nucleus. This process requires poorly understood trafficking of viral particles from the plasma membrane to the endoplasmic reticulum (ER). Once in the ER, host cell chaperones catalyze disulfide bond isomerization and cause the release of a subset of VP1 pentamers from the rest of the capsid, exposing VP2/3 [33]. The residual capsid structure is then transferred into the cytosol by the cellular ER-associated degradation machinery and thence to the nucleus [33,34,35]. This translocation process has been studied thoroughly for the model monkey polyomavirus SV40, which is closely related to JCPyV and BKPyV [34,36,37], but human polyomaviruses also traffic to the ER [38,39]. How the viral genomes then access the nucleus is unclear, but it might involve direct transport through the nuclear pore or cell cycle progression, which results in nuclear envelope breakdown.

Polyomavirus binding to the host cell surface rapidly activates cell signaling cascades by G-protein-coupled receptors (GPCRs) and receptor tyrosine kinases [40,41,42,43,44]. Here, we surveyed the role of cellular signaling proteins in JCPyV infection and found that shRNA- and siRNA-mediated knockdown of phosphoinositide 3’-kinase (PI3K) γ and the PI3Kγ regulatory subunit 5 (PIK3R5) specifically inhibited infection by JCPyV and JC pseudoviruses. Genetic knockout of PI3Kγ or PIK3R5 reduced the ability of JCPyV and several other polyomaviruses viruses to infect cells, a deficit which was rescued by exogenous expression of PI3Kγ. Inhibition of infection was not at the level of cell binding or virus internalization. These findings indicate that activation of PI3Kγ, which couples cell surface binding events to intracellular processes, plays a role in infection by diverse polyomaviruses. These results raise the possibility that inhibitors of PI3Kγ and its signaling cascade may be therapeutically useful.

## 2. Materials and Methods

### 2.1. Cells and Viruses

Human SVG-A glial cells were cultured in MEM basal medium supplemented with 10% fetal bovine serum (FBS), 100 units/mL penicillin-streptomycin, and 10 mM l-glutamine. All other cells were maintained in Dulbecco’s modified Eagle’s medium supplemented with 10% FBS, 100 units/mL penicillin-streptomycin, and 10 mM l-glutamine (DMEM10). Human 293T (ATCC^®^ CRL-3216^™^) was purchased from the American Type Culture Collection, lentiX 293T cells were purchased from ClonTech, and human THP-1 monocytic cells were a gift from Emanuela Bruscia (Yale University).

For all JCPyV infection experiments, we used a previously described lab-adapted strain referred to as Mad-1/SVEΔ [45]. JCPyV was grown in SVG-A cells using 1700 cm^2^ roller bottles in 5% CO_2_ at 37 °C. Infected cells were cultured for 14 days, with the cell culture medium replaced at 7 days. Viral lysates were harvested by scraping cells in the presence of medium. Cells were frozen at −80 °C and thawed in a 37 °C water bath three times. Sodium deoxycholate was added to a final concentration of 0.25%. Samples were centrifuged at 17,000× *g* for 30 min, and supernatant was used for infections. When needed, JCPyV lysates were purified as previously described [39,46]. Briefly, lysates were sonicated three times on ice (50% amplitude 50% duty cycle, power 4, 1 min) and treated with Neuraminidase type II (Sigma, Cat# N6514) at 37 °C for 1 h to release virus from membranes. Samples were subjected to two rounds of Vertrel XF (Fisher Scientific NC9715008) to extract lipids. The viral supernatant was pelleted through a 20% sucrose cushion in a Beckman SW40ti rotor at 150,000× *g* at 4 °C for 3 h. The viral pellet was resuspended in buffer A (10 mM Tris-HCl, 50 mM NaCl, 0.1 mM CaCl_2_) and sonicated three times (30% amplitude 50% duty cycle, power 3, 1 min). The resuspended pellet was loaded onto a CsCl step gradient (1.29–1.35 g/mL) and centrifuged at 115,000× *g* at 4 °C for 18 h in a Beckman SW55ti rotor. The band corresponding to DNA-containing virions was isolated and dialyzed extensively against buffer A. For the virus binding and internalization experiments, CsCl-purified JCPyV was directly labeled with Alexa-Fluor 488 or 633 according to the manufacturer’s protocol (ThermoFisher Scientific, cat# A20000). SV40 strain 776 was prepared in African green monkey CV1 cells as described [47].

JC pseudovirus (JCPsV) was produced as described previously [48]. Briefly, the VP1, VP2, VP3 and pSV40-Cluc (NEB #N0318, expressing *Cypridina* luciferase) plasmids were transfected into HEK293FT cells by using the FuGENE^®^ 6 Transfection Reagent (Promega Madison, WI, USA cat #E2692) in a 3:1:1:2 ratio. The medium was changed on the day following transfection to remove any extracellular transfection reagent. Cells were harvested six days post-transfection, and pseudovirus was purified as described for JCPyV above. Mock pseudovirus controls were generated by transfecting 293FT cells with non-specific DNA and pSV40-Cluc in a 5:1 ratio and following the same production/purification protocol as JCPsV. Similar procedures were used to produce BK polyomavirus and Merkel Cell polyomavirus pseudoviruses expressing *Gaussia* luciferase (BKPsV-Gluc and MCPsV-Gluc, respectively)

### 2.2. Immunoblotting

For immunoblot analyses of PIK3R5, THP-1 cell pools transduced with PIK3R5 shRNA (R2-R5) were compared to matched cells transduced by vector alone (LKO.1). Sub-confluent cultures were lysed with 1× Sample buffer (Bio-Rad, CA, USA), fractionated by SDS-PAGE, and transferred onto PVDF membranes (Millipore, MA, USA). Samples were probed with 1:1000 dilution of rabbit anti-PIK3R5 (Cell Signaling Technologies, Cat#5569) and followed by 1:10,000 dilution of rabbit-specific HRP-conjugated secondary antibody (Jackson ImmunoResearch Laboratories, Inc., Cat#711-035-152). To detect VP1 by immunoblotting, proteins were separated by SDS-PAGE and detected using anti-VP1 PAb597 antibody, which recognizes JCPyV and SV40 VP1 (hybridoma supernatants provided by James Pipas, University of Pittsburgh, Pittsburgh, PA, USA).

### 2.3. Transient shRNA Knockdown and JCPsV Infectivity Assay

SVG-A cells were seeded at 5 × 10^5^ cells per well of a six-well plate. The following day, cells were infected with LKO-based lentiviruses expressing B8 and B9 PIK3R5 shRNAs from the Mission shRNA library (Sigma). Twenty-four h post-transduction, cells were washed twice with phosphate buffered saline (PBS) and incubated in medium containing 10% FBS and 0.5 µg/mL puromycin. As soon as polyclonal puromycin-resistant cells emerged, they were replated and infected 24 h later with 5000 GE of JCPsV for 1 h at 4 °C. After an additional 72 h at 37 °C, culture supernatants were collected, and secreted *Gaussia* Luciferase (GLuc) expression was determined using a BioLux^®^ *Gaussia* Luciferase Assay Kit (New England BioLabs, Ipswich, MA, USA) and a Glomax Discover Luminometer (Promega, Madison, WI, USA).

### 2.4. Stable shRNA Knockdown and JCPsV Infectivity Assay

SVG-A cells transduced with LKO.1 lentivirus or LKO expressing shRNAs targeting PIK3R5 (designated R2 to R5, purchased from Millipore) were selected with puromycin for at least two weeks. Puromycin-resistant cells were plated during exponential growth into 24-well assay dishes at 7500 cells/cm^2^. Cells were mock-infected or infected the next day by replacing standard growth medium with 0.25 mL of a 1:100 dilution of JCPsV containing the *Gaussia* luciferase gene and cultured for 96 h. Culture supernatants were collected at 96 h.p.i., and secreted luciferase activity was assayed as above.

[Relative Light Units (RLU) = RLU (infected)−RLU (mock)]

The sequences of the shRNAs are as follows: R2, TRCN0000033272, CCGGCTGTAGCCAATAAGCTGAGTACTCGAGTACTCAGCTTATTGGCTACAGTTTTTG; R3, TRCN0000195514, CCGGCCCTTACTTTGCCAAAGACTTCTCGAGAAGTCTTTGGCAAAGTAAGGGTTTTTTG; R4, TRCN0000195663, CCGGCTCTCTGTGGAGAACTGAATGCTCGAGCATTCAGTTCTCCACAGAGAGTTTTTTG; R5, TRCN0000300371, CCGGCAGGATCTATAAACTCTTCAACTCGAGTTGAAGAGTTTATAGATCCTGTTTTTG. Replication-defective LKO-based lentivirus were produced in 293T cells by using the packaging plasmids, psPAX2 (Addgene #12260) and pMD2.G (Addgene #12259). Briefly, exponentially growing cells were seeded into 10 cm^2^ dishes and allowed to recover overnight. Five µg of LKO plasmid, psPAX2, and pMD2.G (2:2:1 ratio) were mixed with 10 µg LipoD293 (SignaGen, Frederick, MD, USA) in Opti-MEM and used to transfect 293T cells in Opti-MEM. Cells were supplemented with DMEM + 20% FBS after 4 h. At 48 and 96 h post-transfection, virus supernatant was collected, pooled, and filtered.

### 2.5. Luciferase Activity in SVGA Cells Transfected with Gluc Plasmid

Parental SVG-A cells and PI3Kγ and PIK3R5 knockout cells were transfected with 200 ng of phGluc [49] plasmid DNA using Lipofectamine 2000. Luciferase activity in the culture medium was measured 72 h post-transfection.

### 2.6. siRNA Knockdown and JCPsV Infectivity Assay

siRNAs targeting PIK3R5, DNAJB12, PI 3′-kinase γ, and PI 3′-kinase α as SMARTPools, comprised of multiple hairpins targeting the mRNA, were purchased from Dharmacon (Lafayette, CO, USA) (entrez gene 5294, 54,788, 23,533, and 5290, respectively). Each siRNA pool was transfected into SVG-A cells using the Lipofectamine RNAiMax Reagent according to the manufacturer’s protocol (final siRNA concentration, 20 picomolar). siRNA knockdown was confirmed by qRT-PCR. Forty-eight h post-transfection, cells were infected with 5000 genome equivalents (GE) of JCPsV as described above. Secreted luciferase activity was measured 72 h.p.i. RISC-free siRNA was used as the negative control.

### 2.7. Construction of PI3Kγ and PIK3R5 Knockout Cells

pLentiCRISPRv2 plasmid encoding puromycin resistance was obtained from Addgene (#52961). To replace the puromycin resistance gene with the hygromycin resistance gene, the Agilent QuikChange II XL Site Directed Mutagenesis Kit (#200521) was used insert a HpaI site 3′ to the puromycin resistance gene in pLentiCRISPRv2. Mutagenic primers were obtained from Integrated DNA Technologies: lentiCRISPRv2_ g9959c_F GTTGATTGTCGAGTTAACGCGTTCAGGCACCG and lentiCRISPRv2_ g9959c_R CGGTGCCTGAACGCGTTAACTCGACAATCAAC. The resulting plasmid was digested with BamHI and HpaI to remove the puromycin resistance gene, which was replaced with the hygromycin resistance gene, obtained from Blue Heron Biotech, LLC. (Washington, DC, USA) The sgRNA sequence targeting PIK3R5 (GATCCTGCAGAAGACCCGAG) was obtained from the GeCKOv2 human library [50] and inserted into pLentiCRISPRv2-puro according to the protocol provided by the Zhang lab with minor changes (https://www.addgene.org/static/data/plasmids/52/52961/52961-attachment_B3xTwla0bkYD.pdf). The sgRNA sequence targeting PI3Kγ (ACAACTGCCGAAGGCGCCGG) was designed using a web-based CRISPR design tool (http://crispr.mit.edu) and inserted into pLentiCRISPRv2-hygro. Lentiviruses were produced in HEK293 lentiX cells. Cells were plated in T75 cm^2^ flasks and transfected by using FuGENE HD (Promega) at a 3:1 DNA:FuGENE ratio according to manufacturer’s instructions with the following plasmids: pCMV-dR8.91 (Addgene #2221), the VSV-G envelope encoding plasmid (Addgene #8454/), and pLentiCRISP2v2-sgRNA at a ratio of 3:0.3:3. After 18 h, medium was replaced with 10 mL of DMEM containing 30% FBS. Cells were incubated at 37 °C for an additional 24 h, and lentivirus was harvested every 12 h, for a total of three harvests. Supernatants were combined, filtered using a 0.45 µm pore filter, and either used to infect SVG-A cells immediately or frozen at −80 °C. SVG-A cells at 60% confluence in a T75 cm^2^ flask were infected three times (every 24 h) with 10 mL of the filtered lentivirus supplemented with 8 µg/mL Polybrene (Santa Cruz Biotechnology, Dallas, TX, USA). Cells were then selected with 5 µg/mL puromycin for 3 days or 250 µg/mL hygromycin for 4 days. Single cell isolation and expansion were performed to obtain clonal populations. Genome editing of clonal cell lines was confirmed using the CRISPR deep sequencing service performed at the Center for Computational and Integrative Biology DNA core at the Massachusetts General Hospital.

### 2.8. Infection of CRISPR Knockout Cells with JCPyV

Parental and knockout SVG-A cells were infected in triplicate for 2 h with 150 µL of a 1:40 dilution of CsCl-purified JCPyV. Three days post-infection, cells were fixed in 100% ice cold methanol, permeabilized for 15–20 min in PBS containing 1% triton X100 (PBSTX), and blocked for 1 h at room temperature in 10% Goat Serum in PBS. Cells were incubated for 2 h at 37 °C in 1:50 dilution of PAb597, and then washed three times with PBS. Cells were then incubated for 45 min at room temperature with 200 µL of a 1:1000 dilution of Alexafluor488-labeled anti-mouse antibody, washed three times with PBS, and stained with 1 µg/mL DAPI. Ten fields per well were imaged with Cell Profiler. Additionally, cells were infected with JCPsV expressing secreted *Gaussia* luciferase (JCPsV-Gluc), and the culture medium was assayed for luciferase activity at various times post-infection.

### 2.9. Rescue of JCPyV Infection in PI3Kγ Knockout Cells

Puromycin-resistant pLEX empty vector or pLEX.PI3Kγ, which express transcript variant 1 of the PI3KG-KO3CGgene (NM_002649.3), were packaged in Lenti-X 293T cells as described above. Parental or PI3Kγ knockout (clones Pγ-1 and Pγ-3) SVG-A cells were infected with lentivirus in the presence of 8 µg/mL polybrene and selected with puromycin.

Pooled puromycin-resistant cells were plated in poly-D-lysine-coated 24-well plates and infected in duplicate with 1:200 dilution of JCPyV in serum-free medium for 1 h. Three days post-infection, cells were fixed with 100% ice cold methanol, permeabilized for 10 min in PBSTX, and blocked for 45 min in 10% Goat Serum in PBS. Cells were immunostained for 2.5 h with 1:50 dilution of anti-VP1 PAb597 and then washed three times in PBS. Cells were then incubated with 200 µL Alexafluor488 anti-mouse secondary antibody for 45 min, washed three times with PBS, and stained with DAPI. Fluorescent cells were imaged with a Cell Profiler.

Alternatively, parental SVG-A and PI3Kγ knockout cells (clone Pγ-3) were transduced with LEX.PI3Kγ or empty LEX vector lentivirus and selected in puromycin. Pooled puromycin-resistant cells were plated into 24-well plates at 7500 cells/cm^2^ and infected the next day with 0.25 mL of a 1:100 dilution of JCPyV. Three days later, cells were collected with trypsin and fixed with Cytofix/Cytoperm reagent (BD Biosciences, San Jose, CA, USA). Cells were stained with rabbit polyclonal anti-PI3Kγ (Cell Signaling Technologies, Cat#4252) and anti-VP1 (PAb597), followed by goat anti-rabbit-647 and donkey anti-mouse-488 (Molecular Probes, OR), and analyzed on a BD FACSCalibur flow cytometer. VP1 positivity was determined using mock-infected cell staining and initializing 99.9% of events in the negative (LL) gate. Flow cytometry data were analyzed using FACSDiva and FlowJo software (BD Biosciences, San Jose, CA, USA).

### 2.10. Effect of PI3Kγ Pathway on Infection by Other Polyomaviruses

BKPsV: Parental SVG-A and PI3Kγ knockout SVG-A cells (clone Pγ-3), at 7500 cells/cm^2^ in 24-well dishes, were infected with BKPsV expressing *Gaussia* luciferase. Culture supernatants were collected from cultures at various times post infection and assayed for luciferase activity.

SV40: Parental and PIK3R5 or PI3Kγ knockout SVG-A cells in 24-well plates were infected in triplicate with 150 µL of a 1:40 dilution of CsCl-purified SV40 for 2 h. Forty-eight h.p.i., cells were immunostained for intracellular VP1 with PAb597 as described above, and VP1^+^ cells were enumerated with Cell Profiler.

MCPsV: Parental SVG-A cells or PI3Kγ or PIK3R5 knockout cells were infected with JCPsV-Gluc or Merkel Cell PsV-Gluc at 4 °C for 1 h. Luciferase activity secreted into the culture medium was measured 72 h.p.i. Alternatively, SVG-A cells expressing scrambled control or PIK3R5 shRNA B8 were infected and assayed as above.

### 2.11. Virus Binding and Internalization Studies

Flow cytometry: 24 h prior to binding, 200,000 cells per well were plated in a 12-well dish. Cells were detached using Cell Stripper and washed with chilled PBS, pelleted, and resuspended in chilled PBS containing Alexafluor633-labeled JCPyV or PBS alone. Cells were incubated on ice for 2 h with rocking, washed with chilled PBS, and fixed in 1% paraformaldehyde (PFA). Fluorescence was measured by using BD FACSCalibur (BD Biosciences, San Jose, CA, USA) flow cytometer. Data were analyzed using BD CellQuestPro (BD Biosciences, San Jose, C, USA) and FlowJo software (Tree Star, Inc., Ashland, OR, USA), and cells were gated by size. Experiments were performed in triplicate, and mean and standard error are reported.

To measure internalization, 3.2 × 10^5^ parental SVG-A cells or PIK3R5 knockout cells were plated per compartment in four-compartment 35 mm glass dishes in 2 mL medium in quadruplicate. The next day, cells were prechilled on ice 20 min and Alexafluor633-labeled JCPyV was incubated with cells for 2 h on rocker on ice. For one set of cells, virus was removed and 200 µL 4% PFA was added to cells for 20 min, which were then washed twice with PBS. After 2 h at 0 °C, another set of cells was shifted to 37 °C and virus was allowed to internalize for 1 h in medium containing 10% FBS. Cells were then processed as above. z-stacks were recorded on Zeiss LSM-710 laser scanning confocal microscope, then 10 µL 0.1× trypan blue in PBS was added dropwise. After 15–30 s, a second z-stack of the same fields were recorded. Maximum fluorescence intensity was calculated by using Image J software.

For immunoblot analysis, SVG-A cells or PIK3R5 knockdown cells were inoculated with 5000 GE of JCPsV for 1 h at 4 °C. Cells were washed twice with PBS and either treated with 0.25% trypsin for 10 min at room temperature, left untreated and lysed in RIPA buffer supplemented with protease inhibitors, or incubated at 37 °C for 6 h and then left untreated or treated with trypsin as described above. After cell harvest, samples were heated at 100 °C for 5 min. Proteins were separated by SDS-PAGE, and immunoblotting was performed using anti-VP1 PAb597 antibody.

## 3. Results and Discussion

### 3.1. PIK3R5 and PI3Kγ Knockdown Inhibits JCV Infection

**Figure 1:**
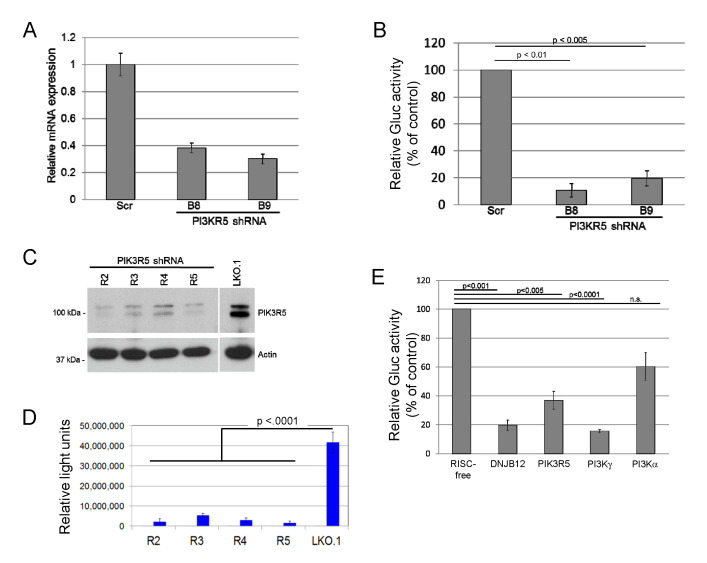
Phosphoinositide 3′-kinase γ (PI3Kγ) knockdown inhibits JC polyomavirus infection. (**A**). SVG-A cells were infected with LKO lentivirus expressing control scrambled shRNA or an shRNA (B8 or B9) targeting PK3IR5 and selected with puromycin. PIK3R5 mRNA levels in puromycin-resistant cells were determined by qRT-PCR. The graph shows average results and standard deviation of three independent experiments. Statistical significance evaluated by two-tailed t test. (**B**). Cells as in panel A were infected with JC pseudovirus (JCPsV) expressing secreted *Gaussia* luciferase (JCPsV-Gluc) at 5000 genome equivalents (GE) per cell. Three days after infection, luciferase activity secreted into the medium was measured. The graph shows average results and standard deviation of three independent experiments, normalized to value for cells that received scrambled shRNA. (**C**). Human THP-1 cells were infected with control LKO.1 lentivirus or LKO.1 expressing one of four different shRNAs targeting PIK3R5 (R2 to R5). Polyclonal cell lines were selected with puromycin and passaged for three weeks. PIK3R5 expression in puromycin-resistant cells was determined by immunoblotting. Actin was used as a loading control. (**D**). SVG-A cells were infected with LKO.1 empty vector or LKO.1 expressing an shRNA targeting PK3IR5 and selected in puromycin. Stably puromycin-resistant cells were infected with JCPsV-Gluc, and luciferase activity secreted into the culture medium was measured three days after infection. The graph shows average results and standard deviation of triplicate determinations. Infectivity of all knockdown cells was statistically significantly lower (*p* < 0.0001) than LKO.1 as assessed by one-way ANOVA. Result is representative of multiple independent experiments. (**E**). SVG-A cells were transfected with siRNAs targeting the indicated genes. Knockdown was of the targeted genes was confirmed by qRT-PCR (data not shown). Two days later, cells were infected with 5000 GE of JCPsV-Gluc, and after an additional 72 h, luciferase activity in the medium was measured. The graph shows average results and standard deviation, normalized to cells that received control RISC-free siRNA.

While surveying the ability of candidate cellular proteins to affect JCPyV entry, we found that transduction of SVG-A cells by JC pseudovirus (JCPsV) was inhibited by shRNAs that targeted PIK3R5, a regulatory subunit of phosphoinositide 3′-kinase γ (PI3Kγ). SVG-A cells, a human glial cell line permissive for JCPyV infection [45], were infected with a lentivirus conferring puromycin resistance and expressing an shRNA targeting one of two independent sequences in PIK3R5 or with a control lentivirus expressing scrambled shRNA. shRNA-mediated knockdown of PIK3R5 mRNA in pooled puromycin-resistant SVG-A cells was confirmed by quantitative RT-PCR (Figure 1A). These cells were then challenged with a JCPsV consisting of a *Gaussia* luciferase (Gluc) reporter plasmid packaged in a JCPyV capsid. Luciferase activity in the culture medium was assayed at 72 h post-infection (h.p.i.) as a measure of JCPsV infectivity. This assay measures events up to expression of encapsidated genes in the nucleus, but not later stages of the virus life cycle. Both PIK3R5 shRNAs inhibited infectivity by 80% or more, compared to the scrambled control (Figure 1B). We then generated a second set of PIK3R5 knockdown cells with independent shRNAs cloned in the LKO.1 lentivirus vector. The available PIK3R5 antibodies were not able to detect the low-level expression of PIK3R5 in parental SVG-A cells, but we used western blotting to document that these shRNAs knocked down PIK3R5 expression in human THP-1 cells, which express abundant PIK3R5 (Figure 1C). In this second set of cells, PIK3R5 knockdown also inhibited JCPsV infectivity by >80% compared to cells transduced by empty LKO.1 vector (Figure 1D). In contrast, the active PIK3R5 shRNA did not inhibit luciferase expression from transfected reporter plasmid DNA (Figure S1), which bypasses the JCPsV entry pathway, suggesting that PIK3R5 facilitated infectious JCPsV entry.

PIK3R5 (also known as p101) is a non-catalytic regulatory subunit of PI3Kγ that stimulates PI3Kγ activity [51]. PIK3R5 does not appear to affect the activity of other PI3K isoforms. Since PIK3R5 knockdown decreases JCPsV infectivity, we hypothesized that knocking down PI3Kγ itself would have a similar effect. Because the PI3Kα catalytic subunit is the most widely expressed and best studied PI3K isoform, we also tested the effect of knocking down PI3Kα. The role of ER chaperone DNAJB12 was also tested, because DNAJB12 is required for infection by the closely related polyomaviruses, SV40 and BK PyV [37]. For these experiments, we transfected SVG-A cells with 20 pmol of siRNA targeting one of the genes or with a RISC-free negative control siRNA. Forty-eight hours after transfection, cells were infected with JCPsV expressing luciferase, and secreted luciferase activity was measured three days later. As shown in Figure 1E, siRNA-mediated knockdown of PIK3R5, PI3Kγ, or DNAJB12 all decreased infectivity by approximately 60 to 80%. Infection was also inhibited by approximately 40% in cells transfected with PI3Kα siRNA, an inhibitory effect that was much less pronounced than that caused by siRNAs targeting PI3Kγ or DNAJB12. These results suggest that PI3Kγ and its regulatory subunit PIK3R5 facilitate infection of SVG-A cells by JCPsV, and that PI3Kα plays a minor role in JCPsV infection compared to PI3Kγ.

### 3.2. PIK3R5 and PI3Kγ Knockout Inhibits Infection by JCPsV

**Figure 2:**
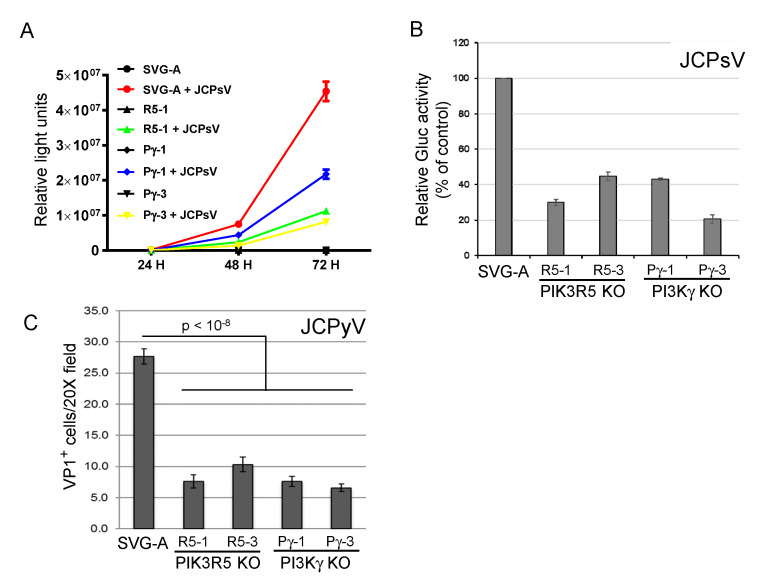
PI3Kγ and PIK3R5 knockout inhibits JC polyomavirus infection. (**A**). Parental SVG-A cells or PI3Kγ (Pγ-1 and Pγ-3) or PIK3R5 (R5-1) knockout cells were mock-infected or infected with JCPsV-Gluc. At the indicated times post-infection, luciferase activity in the medium was measured in triplicate. (**B**). Parental SVG-A cells or PI3Kγ or PIK3R5 knockout (KO) cells were infected with JCPsV-Gluc. Luciferase activity in the culture medium was measured three days after infection and normalized to control infected cells. (**C**). Parental SVG-A cells and PI3Kγ or PIK3R5 knockout (KO) cells were infected with JCPyV. Cells were fixed and stained for VP1 three days post-infection. VP1 positive cells are expressed as number of VP1 positive cells per 20× microscope field plus and minus standard error. Infectivity of all knockouts was statistically significantly lower than SVG-A cells as assessed by 2-tailed *t*-test with equal variance.

To confirm the role of the PI3Kγ pathway in JCPyV infection, we used the CRISPR/Cas9 system to generate clonal PIK3R5 and PI3Kγ knock-out SVG-A cells. Gene knock-out was confirmed by sequencing the targeted gene in each cell line (Figure S2). In two independent cell lines knocked out for either gene (*PIK3R5*, R5-1, and R5-3; *PI3Kγ*, Pγ-1, and Pγ-3), JCPsV infectivity was reduced by 50–80%, as assessed by secreted luciferase activity (Figure 2A,B). We also infected the knock-out cells with authentic JCPyV and assessed infection 72 h.p.i. by immunofluorescence staining to detect newly synthesized VP1 expressed by virus that had reached the nucleus. As was the case for JCPsV, knockout of either gene caused an approximately 50% reduction in JCPyV infectivity (Figure 2C). These results confirm that the PI3Kγ pathway is important but not essential for JCV infection. Either PI3Kγ is not the sole pathway that can support JCV infection in these cells, or during the derivation of the clonal knock-out cells, compensatory changes occurred in the cells that partially abrogated the PI3Kγ requirement.

### 3.3. PI3Kγ Expression Rescues JCPyV Infection in Knock-Out Cells

**Figure 3:**
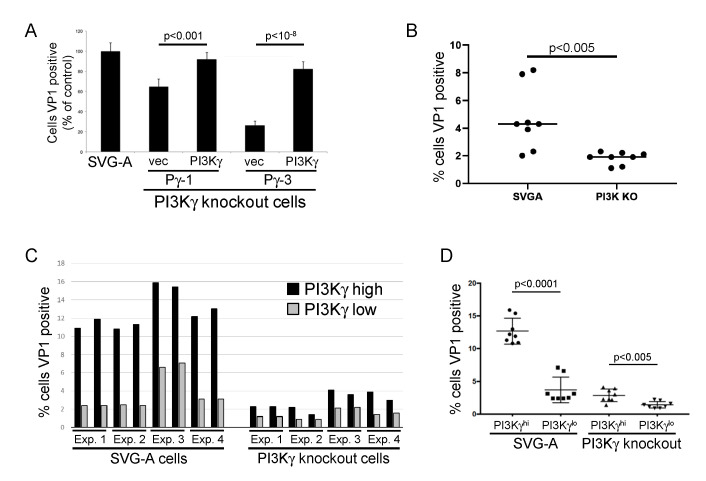
PI3Kγ expression rescues JC polyomavirus infection defect caused by PI3Kγ knockout. (**A**). PI3Kγ knockout SVG-A cells were infected with LEX-PI3Kγ or empty LEX vector (vec) lentivirus and selected in medium containing puromycin. Puromycin-resistant cells were infected with JCPyV. Three days later, cells were immunostained for intracellular VP1. Graph shows average fraction of cells that expressed VP1, as assessed by Cell Profiler, normalized to infected parental SVG-A cells, plus and minus standard error. Infectivity of cells with transduced *PI3Kγ* was statistically significantly higher than cells transduced with empty vector as assessed by 2-tailed t-test with equal variance. In a typical experiment, ~1% of infected control cells were VP1^+^. (**B**). Parental SVG-A cells and PI3Kγ knockout cells (clone Pγ-3) were transduced with empty LEX lentivirus and selected in puromycin. Pooled puromycin-resistant cells were infected with JCPyV virus. Cells were fixed and immunostained for intracellular VP1 three days later, and VP1 expression was analyzed by flow cytometry. Graph shows fraction of cells that expressed VP1. Results are aggregated data from four independent experiments, each performed in duplicate. Representative primary data are shown in Figure S3. (**C**). Parental SVG-A cells and PI3Kγ knockout cells (clone Pγ-3) were infected in duplicate with LEX-PI3Kγ vector lentivirus and selected in puromycin. Pooled puromycin-resistant cells were infected with JCPyV, stained for intracellular VP1 and PI3Kγ three days later, and assessed by flow cytometry. Graph shows the percentage of cells expressing VP1 after infection with JCPyV in PI3Kγ^hi^ (black bars) and PI3Kγ^lo^ (grey bars) cell populations. (**D**). The graph shows the percentage of cells expressing VP1 after infection with JC polyomavirus (JCPyV) as in panel **C** in the PI3Kγ^hi^ and PI3Kγ^lo^ populations, plus and minus standard deviation. Each point represents an individual determination. For panels **B**, **C**, and **D**, representative primary data are shown in Figure S3. Data and standard deviation intervals are tabulated in Table 1.

We performed gene rescue experiments to formally establish the requirement for PI3Kγ in JCPyV infection. The wild-type *PI3Kγ* gene was cloned into the pLEX lentivirus vector, which confers puromycin resistance, and its ability to rescue infection was tested in PI3Kγ knock-out cells. We first used imaging to assess rescue. We infected control PI3Kγ knockout cells and the PI3Kγ-reconstituted cells with infectious JCPyV and stained with anti-VP1 antibody three days later. An automated fluorescent microscopy imaging system was used to score the cells for VP1 expression. As shown in Figure 3A, transduction of the *PI3Kγ* gene into either of the two PI3Kγ knockout cells restored the fraction of cells expressing VP1 to nearly control levels.

We also used flow cytometry to assess the effect of exogenous PI3Kγ in four independent JCPyV infection experiments, each conducted in duplicate. Three experiments were performed in one set of matched cell lines, the other in an independent set of matched cell lines. We transduced parental SVG-A cells and PI3Kγ knockout cells with empty LEX vector or LEX-PI3Kγ lentivirus and selected with puromycin. We then infected pooled, puromycin-resistant cells with infectious JCPyV and measured PI3Kγ and VP1 expression by flow cytometry at 72 h.p.i. (Figure S3). Figure 3B shows that in the absence of exogenous PI3Kγ, infectivity in the PI3Kγ knockout cells was reduced by ~60% compared to parental SVG-A cells (1.8% cells infected vs. 4.7%), confirming the dependence on PI3Kγ described above. Introduction of the *PI3Kγ* gene resulted in increased PI3Kγ expression in approximately one-third of the cells (Figure S3). We examined the fraction of cells expressing VP1 in PI3Kγ^hi^ cells, compared to PI3Kγ^lo^ cells. In all four experiments, a higher fraction of cells in the PI3Kγ^hi^ population expressed VP1, compared to the PI3Kγ^lo^ cells (Figure 3C,D and Table 1). This comparison internally controls for any minor differences in cell density or JCPsV titer, among other factors. In parental SVG-A cells, approximately four-fold more PI3Kγ^hi^ cells were infected by JCPyV than were PI3Kγ^lo^ cells, implying that PI3Kγ expression is limiting for infection, even in wild-type cells. A similar but muted trend was seen in the PI3Kγ knockout cells, which showed two-fold higher infection in the PI3Kγ^hi^ cells. As noted above, the knockout cells may have adapted during their selection and clonal outgrowth, resulting in the up- or down-regulation of other proteins or lipids that influence PI3K pathway homeostasis or other processes that affect JCPyV infectivity. Taken together, these experiments establish that PI3Kγ facilitates infection of SVG-A cells by JCPsV and JCPyV but is not absolutely required for infection.

### 3.4. PI3Kγ Facilitates Infection by other Polyomaviruses

**Figure 4:**
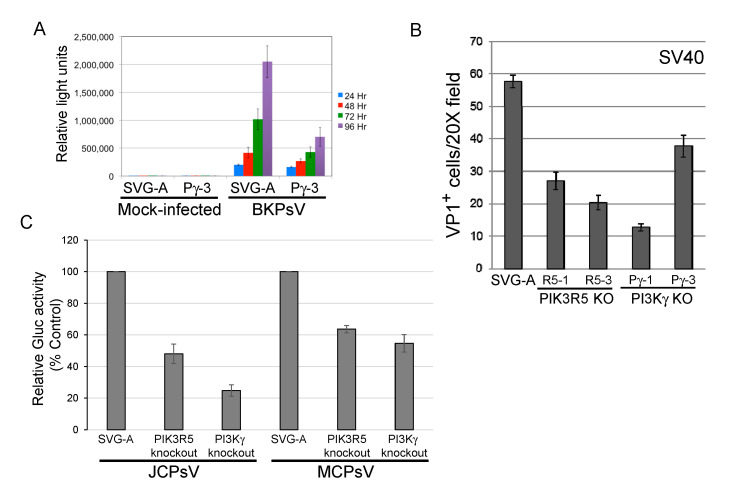
PI3Kγ pathway facilitates infection by other polyomaviruses. (**A**). Parental SVG-A cells and PI3Kγ knockout (clone Pγ-3) cells were mock-infected or infected with BKPsV expressing secreted *Gaussia* luciferase. At the indicated times after infection, luciferase activity in the culture medium was measured and expressed as relative light units. Results show average plus and minus standard deviation of triplicate measurements, and are representative of multiple different experiments. (**B**). Parental and knockout SVG-A cells were infected with SV40. Three days post-infection, cells were fixed and immunostained for intracellular VP1. VP1 positive cells were counted by indirect immunofluorescence. Results show average and standard error of multiple experiments. (**C**). Parental SVG-A cells or PIK3R5 or PI3Kγ knockout cells were infected for 1 h at 4 °C with JCPsV-Gluc or MCPsV-GLuc. Three days post-infection, luciferase activity in the culture medium was measured. Graph shows average results of triplicate infections plus and minus standard deviation, expressed as activity normalized to cells without knockout.

We next determined whether the PI3Kγ pathway facilitated infection by other polyomaviruses. PI3Kγ knockout reduced transduction by BKPsV by approximately 2–3-fold (Figure 4A), similar to the results with JCPyV or JCPsV. Similarly, SV40 infectivity was reduced by approximately 50% in SVG-A cells knocked out for PI3Kγ or PIK3R5, as assessed by flow cytometry for expression of large T antigen, the major polyomavirus early protein (Figure 4B). Merkel cell polyomavirus is more distantly related to JCPyV, and knockout of PIK3R5 or PI3Kγ in SVG-A cells inhibited transduction by MCPsV by approximately 40–50% (Figure 4C), as did shRNA-mediated knockdown of PIK3R5 (Figure S4). These results show that multiple human and primate polyomaviruses require the PI3Kγ pathway for efficient infection of SVG-A cells.

### 3.5. JCPyV Binding and Internalization Is not Inhibited in PIK3R5 Knockout SVG-A Cells

**Figure 5:**
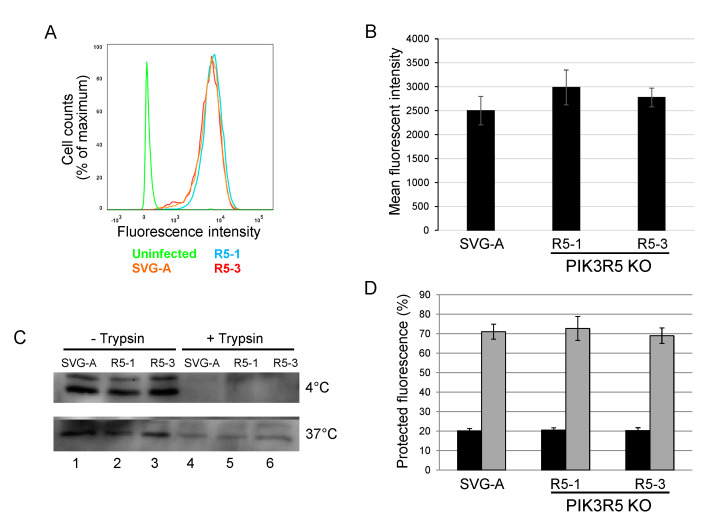
PI3Kγ is not required for JC polyomavirus binding or internalization. (**A**). Parental SVG-A cells and PI3Kγ knockout cells (clones R5-1 and R5-3) were incubated with Alexafluor633-labeled JCPyV on ice for 2 h and fixed in 1% PFA. Fluorescence intensity was measured by flow cytometry and shown as a histogram. Fluorescence of cells not exposed to virus is shown in green. (**B**). Quantitation of experiments as in panel A. Average and standard error of three independent experiments are shown. (**C**). Western blot analysis of virus binding and internalization. Parental SVG-A cells or PIK3R5 knockout cells (clones R5-1 and R5-3) were incubated with 5000 GE JCPsV for 1 h at 4 °C. One set of cells (top panel) was immediately processed by direct lysis or lysis after incubation in 0.25% trypsin. Another set of cells (bottom panel) was shifted to 37 °C and harvested 6 h.p.i. by direct lysis or trypsin treatment as above. (**D**). Parental SVG-A cells and PIK3R5 knockout SVG-A cells were incubated with Alexafluor633-labeled JCPsV for 1 h on ice and harvested (black bars). After 2 h at 0 °C, cells were shifted to 37 °C and virus was allowed to internalize for 1 h prior to analysis (grey bars). After incubation, cells were fixed and imaged by confocal microscopy before and after trypan blue addition. Graph shows fraction of fluorescence that was protected from trypan blue-induced quenching. Maximum fluorescence intensity was calculated by using Image J software.

To begin to explore the step of infection that is facilitated by the PI3Kγ pathway, we tested whether JCPyV binding and internalization were affected by PIK3R5 knockout. Parental or PIK3R5 knockout SVG-A cells were incubated with AlexaFluor488-labeled JCPyV for one hour at 4 °C (to block endocytosis). Cells were then washed and analyzed by flow cytometry to determine the levels of cell-associated virus. The levels of fluorescence were indistinguishable between parental SVG-A cells and the PIK3R5 knockout cell lines, showing that binding of JCPyV to cells was not inhibited by PIK3R5 knockout (Figure 5A,B). As an independent measure of binding, unlabeled JCPsV was incubated with parental SVG-A and PIK3R5 knockout cells for one hour at 4 °C. After extensive washing, cells were harvested, lysed, and subjected to electrophoresis and immunoblotting for VP1. As shown in Figure 5C, upper panel, lanes 1, 2, and 3, similar amounts of VP1 bound to cells whether or not they expressed PIK3R5. This signal was eliminated by cell surface trypsin treatment prior to cell harvest, as expected (Figure 5C, upper panel, lanes 4, 5, and 6).

We also determined if the PI3Kγ pathway facilitated virus internalization. PIK3R5 knockout cells were exposed to AlexaFluor488-labeled JCPyV at 0 °C and treated with trypan blue, a membrane-impermeable chemical that quenches the fluorescent signal of surface-bound virus particle but has no effect on internalized virus. Fluorescence was then analyzed by confocal microscopy. After two h.p.i. at 0 °C, the infected parental and the two knockout cell lines showed no difference in fluorescent signal, with approximately 20% of input fluorescence being resistant to trypan blue, confirming that most of the virus was not internalized under these conditions (Figure 5D, black bars). Cells exposed to labeled viruses at 0 °C were then shifted to 37 °C to initiate endocytosis and examined one hour later by confocal microscopy before and after trypan blue addition. Under these conditions, approximately 70% of virus was internalized, as assessed by fluorescent signal resistant to quenching by trypan blue (Figure 5D, grey bars). There was no difference in the amount of internalized virus between the parental cells and the two knockout cell lines, indicating that PIK3R5 knockout did not affect internalization. Similarly, western blotting showed that parental and knockout cells displayed comparable levels of internalized (i.e., trypsin-resistant) VP1 after six hours at 37 °C (Figure 5C, bottom panel, lanes 4–6). Taken together, these data show that JCPyV binding to cells and virus internalization do not require PIK3R5. Thus, PI3Kγ appears to facilitate execution of one or more intracellular steps in polyomavirus entry, perhaps capsid disassembly or trafficking of the virus to the ER or nucleus.

## 4. Conclusions

These experiments were conducted with the goal of identifying cell proteins required for infection by pathogenic human polyomaviruses that may be amenable to inhibition as a therapeutic strategy. By testing the role of targetable proteins comprising intracellular signaling pathways and using an assay for the early events of JC virus infection, we showed that the PI3′-kinase γ pathway facilitates infection of human glial cells via a number of different polyomaviruses. It is known that binding of polyomavirus virions to the cell surface stimulates signaling required for efficient infection and that PI3Kγ couples GPCR activation to intracellular signaling events. We speculate that virion-triggered GPCR signaling is transmitted by PI3Kγ to facilitate an early step in JC virus infection, perhaps capsid disassembly or trafficking of the virus or viral DNA to the ER or nucleus. It may be possible to repurpose anti-cancer drugs targeting the PI3K pathway, such as Duvelisib (a dual PI3K-γ and PI3Kδ inhibitor), as novel anti-viral agents. However, the inhibitory effects of disrupting PI3Kγ are relatively modest, suggesting that agents that solely inhibit this pathway are unlikely to be sufficient to impose a stringent control on polyomavirus infection, although they might be useful components in combination anti-viral therapy. In addition, further study of the role of PI3Kγ in polyomavirus entry may reveal additional mechanistic information about this process and identify additional anti-viral targets.

## Figures and Tables

**Table 1:** Percentage of VP1+ Cells.

	SVGA	SVGA + PI3K			PI3K KO	PI3K KO + PI3K		
		High	Low	High/Low		High	Low	High/Low
Exp. 1a *	4.3	10.9	2.4	4.54	1.9	2.3	1.2	1.92
Exp. 1b	4.4	11.9	2.4	4.96	1.9	2.3	1.1	2.09
Exp. 2a	2.3	10.8	2.5	4.32	1.2	2.2	0.9	2.44
Exp. 2b	2.0	11.3	2.4	4.71	1.1	1.4	0.9	1.56
Exp. 3a	7.9	15.9	6.6	2.41	2.1	4.1	2.1	1.95
Exp. 3b	8.2	15.4	7.1	2.17	2.2	3.6	2.2	1.64
Exp. 4a	3.9	12.2	3.1	3.94	2.3	3.9	1.4	2.79
Exp. 4b	4.3	13.0	3.1	4.12	1.9	3.0	1.6	1.88
Average	4.7 ± 2.3			3.9 ± 1.04	1.8 ± 0.44	2.03 ± 0.41		

* Experiments 1, 2, and 3 were performed in duplicate with one set of cell lines. Experiment 4 was performed in duplicate with an independently-derived set of cell lines.

## Supplementary Materials

The following are available online at https://www.mdpi.com/1999-4915/12/10/1190/s1, Figure S1: Luciferase expression is not reduced in transfected PIK3R5 and PI3Kγ knockout SVG-A cells; Figure S2: Sequences of targeted genes in knockout cell lines; Figure S3: Representative two-dimensional flow cytometry histograms for PI3Kγ rescue experiment; Figure S4: PIK3R5 knockdown cells are refractory to infection by Merkel Cell Polyomavirus.
