# Supplementary material for: Phosphoinositide 3′-Kinase γ Facilitates Polyomavirus Infection
Source: Viruses. 2020 Oct 20;12(10):1190. doi: 10.3390/v12101190 (PMC7589550; doi:10.3390/v12101190)
Supplement: Supplementary file 1 [file viruses-12-01190-s001.pdf]

Supplementary Information

Figure S1. Luciferase expression is not reduced in transfected PIK3R5 and PI3Kγ knockout SVG-A cells. Parental SVG-A cells and PI3Kγ and PIK3R5 knockout (KO) cells were transfected with phGluc plasmid DNA. Luciferase activity was measured in the medium 72 h post-transfection.

Figure S2. Sequences of targeted genes in knockout cell lines. Sequences surrounding the sgRNA binding sites are shown. The top line in each set shows the wild-type sequence with the sgRNA binding site boxed in red. The other lines show the sequences obtained from deep sequencing of cloned knockout cell lines. Note that PIK3R5 knockout cell line R5-3 has a single mutant cell strain; the other knockout cells have two mutant cell strains, each with a different mutation. No wild-type sequences were observed in the cloned knockout cell lines.

Figure S3. Representative two-dimensional flow cytometry histograms for PI3Kγ rescue experiment. Parental SVG-A cell and PI3Kγ knockout (clone Pγ-3) SVG-A cells were infected with LEX-PI3Kγ or empty LEX vector lentivirus and selected in puromycin. Pooled puromycin-resistant cells were mock-infected or infected in duplicate with JCPyV. Three days later, cells were fixed, immunostained for intracellular VP1 and PI3Kγ and assessed by flow cytometry. Panels show two-dimensional histograms. The percentage of cells that are VP1 positive in the PI3Kγ^{hi} and PI3Kγ^{lo} quadrants in the infected samples are shown in the red boxes.

Figure S4. PIK3R5 knockdown cells are refractory to infection by Merkel Cell Polyomavirus. SVG-A cells were transduced with scrambled control shRNA or shRNA (clone

B8) and selected with puromycin. Puromycin-resistant cells were infected with the indicated number of genome equivalents of JCPsV and MCPsV expressing *Gaussia* luciferase. Gluc activity in the culture medium was measured 72 h.p.i.

Fig. S1

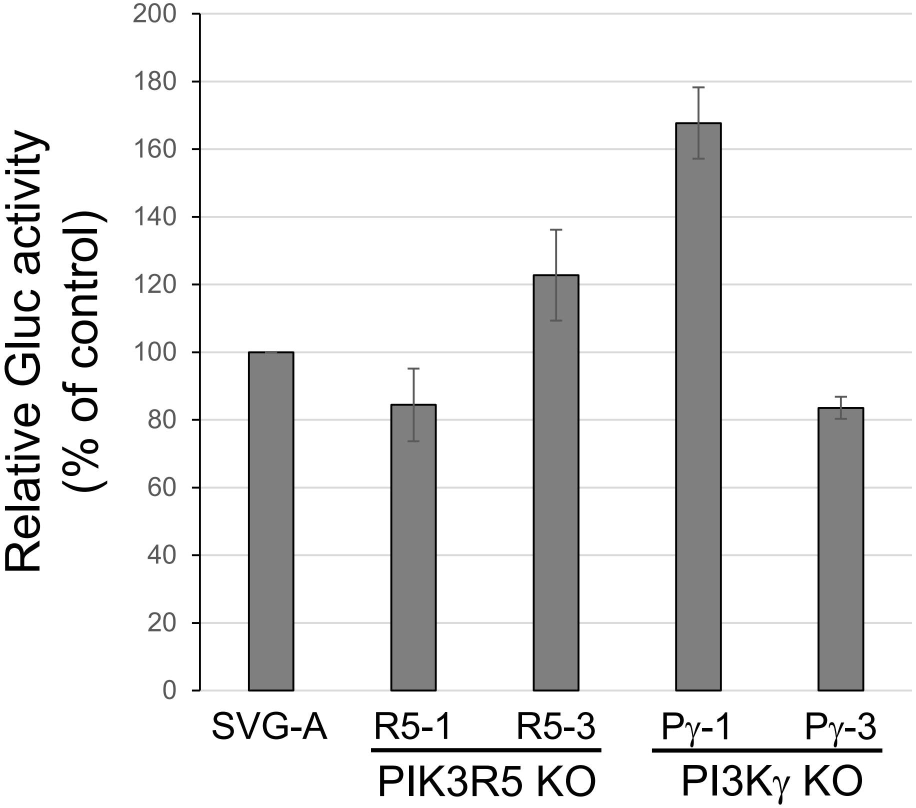

Relative Gluc activity
(% of control)
200
180
160
140
120
100
80
60
40
20
0
SVG-A
R5-1
R5-3
Pγ-1
Pγ-3
PIK3R5 KO
PI3Kγ KO

Fig. S2

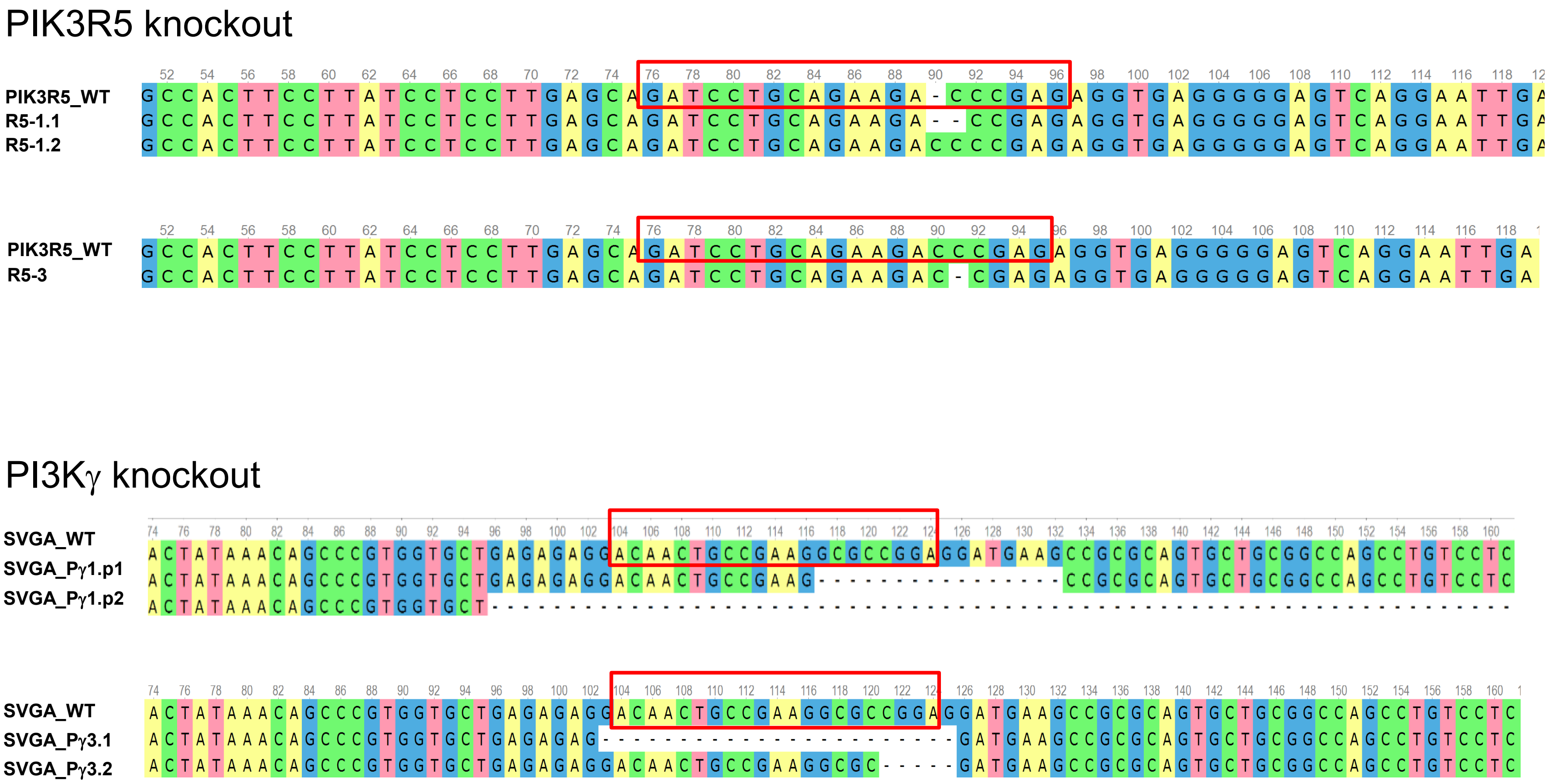
PIK3R5 knockout
PIK3R5_WT
R5-1.1
R5-1.2
PIK3R5_WT
R5-3
PI3Kγ knockout
SVGA_WT
SVGA_Pγ1.p1
SVGA_Pγ1.p2
SVGA_WT
SVGA_Pγ3.1
SVGA_Pγ3.2

Fig. S3

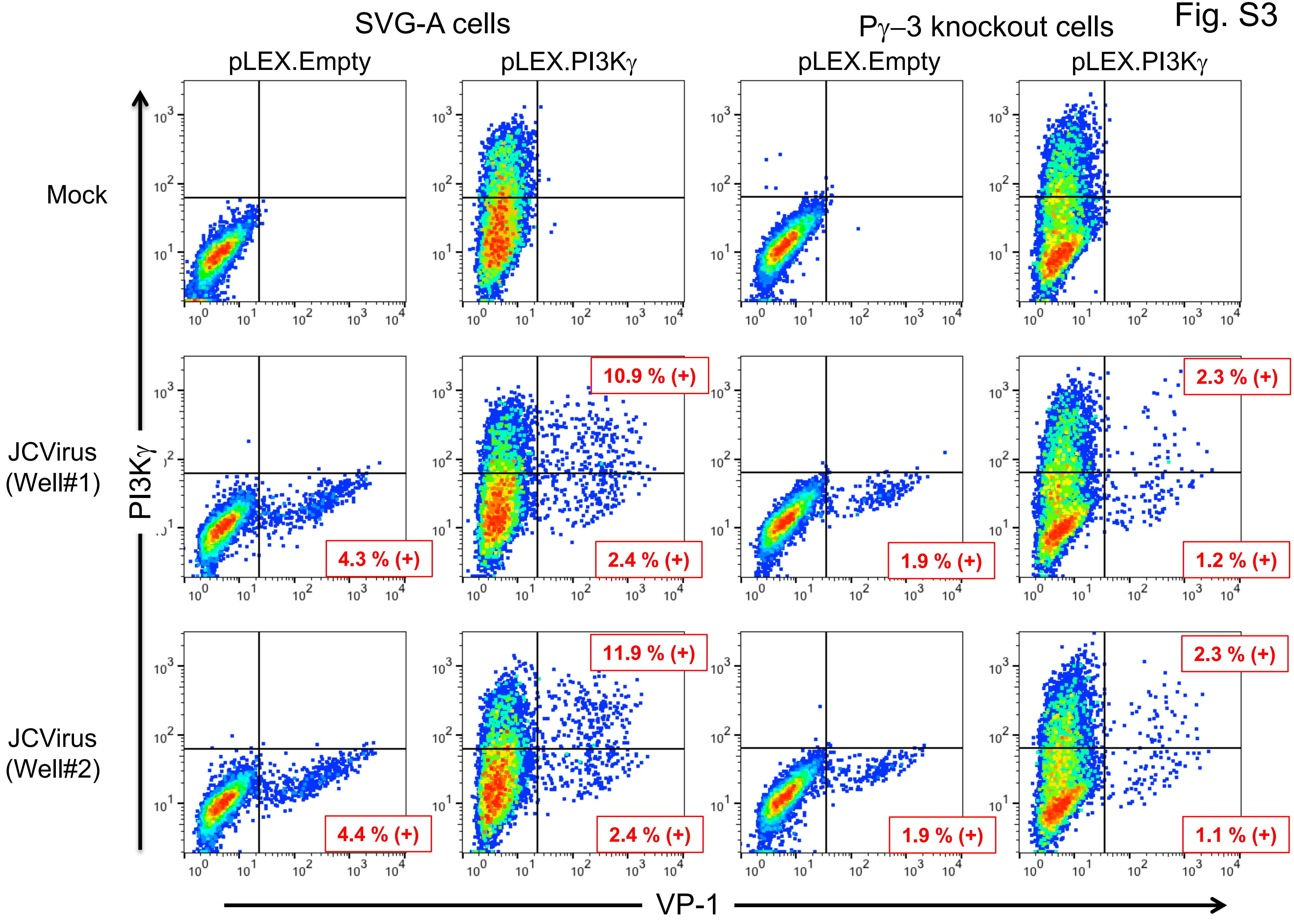
SVG-A cells
Pγ–3 knockout cells
pLEX.Empty
pLEX.PI3Kγ
pLEX.Empty
pLEX.PI3Kγ
Mock
JCVirus (Well#1)
JCVirus (Well#2)
PI3Kγ
VP-1
10.9 % (+)
4.3 % (+)
2.4 % (+)
1.9 % (+)
2.3 % (+)
1.2 % (+)
11.9 % (+)
4.4 % (+)
2.4 % (+)
1.9 % (+)
2.3 % (+)
1.1 % (+)

Fig. S4

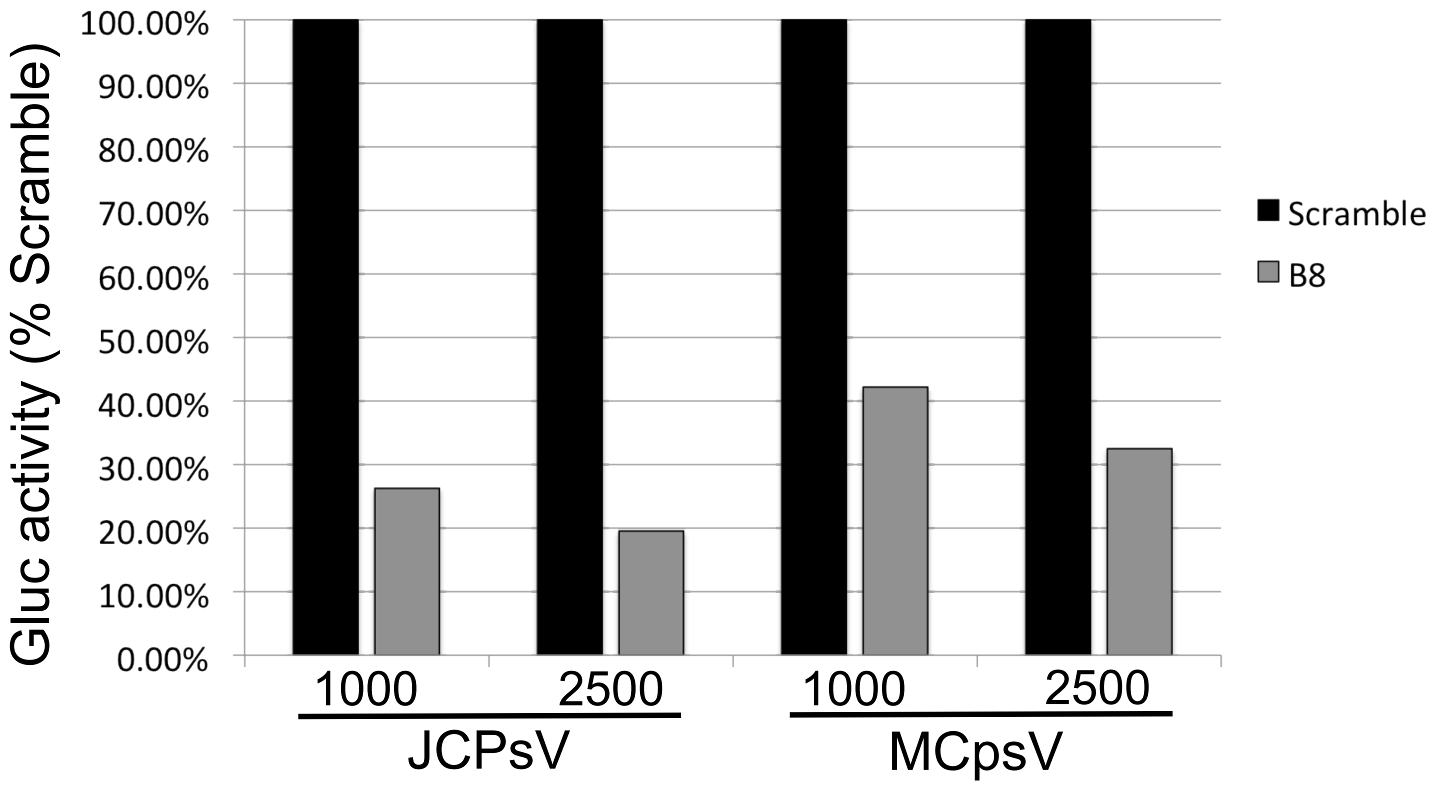
Gluc activity (% Scramble)
100.00%
90.00%
80.00%
70.00%
60.00%
50.00%
40.00%
30.00%
20.00%
10.00%
0.00%
1000
2500
1000
2500
JCPsV
MCpsV
Scramble
B8
